# Efficacy Comparison of Tenofovir and Entecavir in HBeAg-Positive Chronic Hepatitis B Patients with High HBV DNA

**DOI:** 10.1155/2016/6725073

**Published:** 2016-03-01

**Authors:** Hong Shi, Mingxing Huang, Guoli Lin, Xiangyong Li, Yuankai Wu, Yusheng Jie, Yutian Chong

**Affiliations:** ^1^Department of Infectious Disease, Third Affiliated Hospital of Sun Yat-sen University, Guangzhou 510630, China; ^2^Department of Infectious Disease, Fifth Affiliated Hospital of Sun Yat-sen University, Zhuhai 519000, China

## Abstract

*Objectives*. To compare entecavir (ETV) and tenofovir disoproxil fumarate (TDF) effects in chronic hepatitis B (CHB) patients with high HBV DNA.* Method*. 96 patients treated initially with tenofovir (TDF group) or entecavir (ETV group) were included in this retrospective study. The following parameters were assessed: HBeAg and hepatitis B e antibody (anti-HBe) status, serum alanine aminotransferase (ALT), and HBV-DNA levels at weeks 4, 12, 24, 36, 48, 60, 72, and 96; time to ALT normalization, undetectable HBV-DNA levels, and HBeAg seroconversion; total duration of follow-up and adverse reactions.* Results*. The patients included 66 (69%) and 30 (31%) individuals administered ETV and TDF, respectively, comprising 75% males. They were 35.1 ± 4.5 and 33.7 ± 4.6 years old in ETV and TDF groups, respectively. At 36 weeks, the response rate was significantly higher in the TDF group than in ETV treated patients (90% versus 69.7%, *p* = 0.03). At 48 weeks, less patients administered ETV showed undetectable HBV-DNA levels compared with the TDF group (86.4% versus 96.7%), a non-statistically significant difference (*p* = 0.13). Only 1 ETV treated patient developed virological breakthrough at 48–96 w. No adverse reactions were found.* Conclusion*. ETV and TDF are comparable in efficacy and safety to suppress HBV-DNA replication in HBeAg-positive CHB patients with high HBV DNA.

## 1. Introduction

Chronic hepatitis B virus (HBV) infection is a significant health problem worldwide; it may cause serious complications such as cirrhosis, liver failure, and hepatocellular carcinoma (HCC) [1]. In China, although HBV prevalence was reduced to 7.2% in the general population by 2006, 97 million people are HBV carriers, with at least 20 million still suffering from active chronic HBV infection, alone or in combination with cirrhosis and/or HCC [2]. Early diagnosis and treatment of chronic hepatitis B (CHB) infection is crucial for reducing morbidity and mortality.

The primary goal of CHB treatment is to reduce the risk of developing chronic liver disease and associated complications. Two different treatment strategies are commonly used for patients with CHB infection: therapy of fixed duration with immunomodulators such as standard or PEGylated interferon-*α*, long-term treatment with the nucleos(t)ide analogues lamivudine (LAM), adefovir dipivoxil (ADV), entecavir (ETV), telbivudine (LdT), or tenofovir (TDF). Rates of resistance to LAM and ADV have been reported to be 65–70% and 18–29%, respectively, after 4-5 years of treatment [3, 4]. LdT results in 5–25% resistance in HBeAg-positive patients, with 2.3–11% resistance obtained in HBeAg-negative patients [5]. ETV and TDF are recommended first-line therapeutics for CHB in current guidelines thanks to their high potency in viral suppression, providing also genetic barriers against resistance [6–8].

A few studies have compared the efficacy of ETV and TDF. For instance, we previously demonstrated that TDF and ETV both rapidly inhibit HBV DNA replication in naïve CHB patients [9]. However, data concerning CHB patients with high HBV-DNA are limited. The aim of the current study was to investigate the effects of ETV and TDF in HBeAg-positive CHB patients with high HBV DNA.

## 2. Materials and Methods

### 2.1. Study Design and Data Collection

This was a retrospective cohort study, conducted at the Third Affiliated Hospital of Sun Yat-sen University, from March 2012 to March 2015. A total of 96 HBeAg positive CHB patients were assessed, including 66 and 30 cases treated orally with entecavir at 0.5 mg/day (ETV group) and tenofovir at 300 mg/day (TDF group). TDF and ETV, both first line therapeutics for HBeAg-positive chronic hepatitis B patients with high HBV DNA, were presented to the subjects for their selection in clinic. The study was approved by the ethics committee of the Third Affiliated Hospital of Sun Yat-sen University.

Inclusion criteria were (1) diagnosis of CHB with no prior history of treatment for CHB, including TDF and ETV administration; (2) HBeAg seropositivity; (3) pretreatment serum HBV-DNA levels > 10^6^ IU/mL; (4) at least 18 years of age at treatment initiation; (5) treatment for at least one year. Patients were excluded from the study if they (1) were coinfected with HCV, HDV, and/or HIV; (2) had comorbidities with alcoholic, drug-induced, or autoimmune liver diseases.

Parameters such as age, height, weight, serum alanine aminotransferase (ALT) and HBV DNA levels at baseline, gender, alcohol use, smoking status, were recorded for each patient prior to treatment. The following parameters were assessed: HBeAg and anti-HBe status, serum ALT and HBV-DNA levels at weeks 4, 12, 24, 36, 48, 60, 72, and 96 of treatment; time to ALT normalization, undetectable HBV-DNA levels, and HBeAg seroconversion; total duration of follow-up. Independent variables reflecting the virological response to treatment were determined by survival analysis. Cumulative probability of virological response was assessed in patients treated with entecavir and tenofovir, respectively. Compliance with therapy was evaluated.

### 2.2. Definitions

Complete viral suppression was defined as undetectable serum HBV DNA (<100 IU/mL, or below the lower limit of quantification of the PCR assay) at week 48. Virological breakthrough was defined as a >1 log_10_ IU/mL increase in serum HBV-DNA levels from nadir in two consecutive measurements.

ALT ≤ 40 U/L was considered normal [10].

### 2.3. Statistical Analyses

The SPSS 13.0 software (SPSS Inc., Chicago, IL, USA) was used for all statistical analyses. Categorical variables were defined as proportion (%) and compared by Chi-square or Fisher's exact test. Continuous variables are mean ± standard deviation (SD) and were assessed by Student's *t*-test or Mann-Whitney *U* test, as appropriate. Cox regression analysis was performed in search of variables determining the virological response. Cumulative rates of complete viral suppression were analyzed by the Kaplan-Meier method. *p* < 0.05 was considered statistically significant.

## 3. Results

### 3.1. Patient Characteristics

A total of 96 patients were included in this study, comprising 66 and 30 cases treated with entecavir (ETV group) and tenofovir (TDF group), respectively. They were 35.1 ± 4.5 and 33.7 ± 4.6 years old in the ETV and TDF groups, respectively. No statistically significant differences were observed between the ETV and TDF groups patients in age, gender, height, weight, smoking and drinking history, HBV family history, baseline ALT levels, and HBV-DNA levels (Table 1).

### 3.2. ALT Normalization Rates

**Figure 1:**
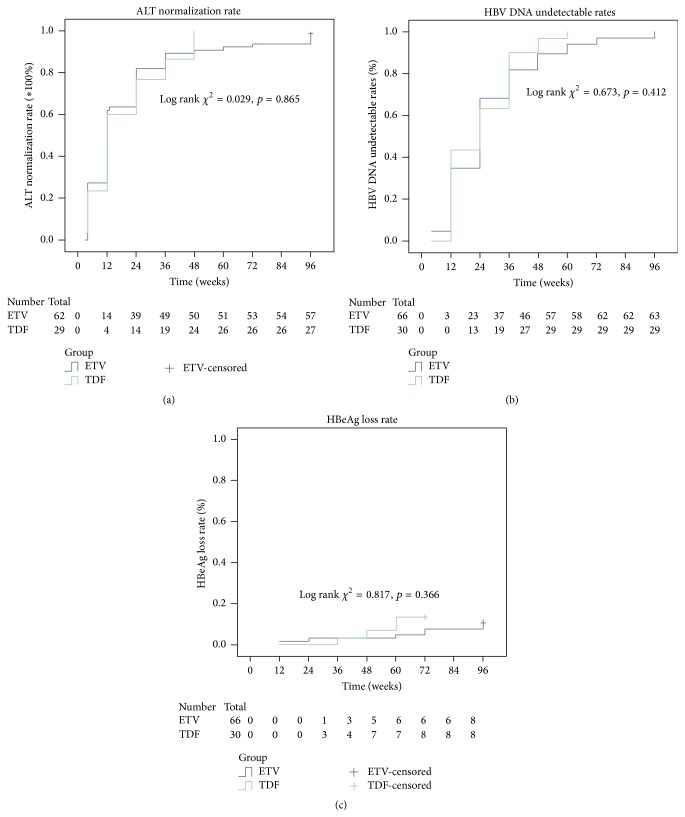
Kaplan-Meier analyses. (a) Alanine aminotransferase (ALT) normalization rates; (b) HBV undetectable DNA rates; (c) HBeAg seroconversion rates. TDF, tenofovir; ETV, entecavir.

The fractions of patients with normalized serum ALT levels at weeks 4, 12, 24, 36, 48, and 72 did not differ significantly between the two groups. However, serum ALT levels in ETV treated individuals were significantly lower compared with values obtained for the TDF group at 12 and 24 weeks (Table 2). The Kaplan-Meier survival analysis showed no statistically significant difference in the normalization rates between the two groups (Figure 1(a)).

### 3.3. Virological Response

**Figure 2:**
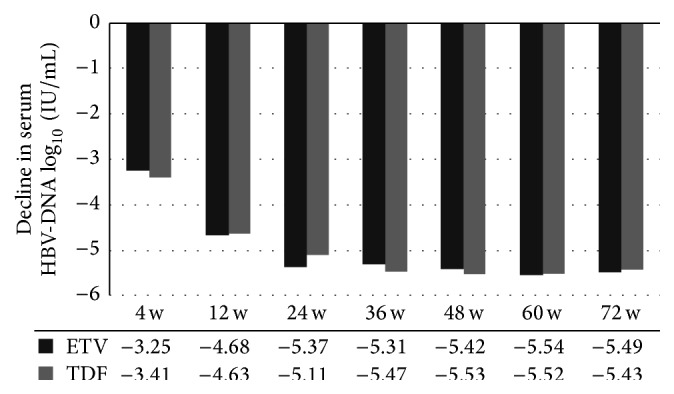
Decline of serum HBV DNA levels from baseline values. TDF, tenofovir; ETV, entecavir; IU/mL, international unit per milliliter; w, weeks.

Patients treated with ETV and TDF had high viral response rates at 48 weeks (86.4% versus 96.7%), with a higher value in the TDF group, although the difference was not statistically significant (*p* = 0.13). At 36 w, the response rate in the TDF group was significantly higher than that obtained for ETV treated patients (90% versus 69.7%, *p* = 0.03) (Table 2). At 4, 12, 24, and 48 weeks HBV-DNA levels were similar between the two groups, with no statistically significant difference (Table 2). The decline of serum HBV DNA from baseline values showed no statistically significant difference between the two groups at any time point (Figure 2). In addition, Kaplan-Meier survival analysis revealed no significant difference in undetectable HBV-DNA rates between the two treatment groups (Figure 1(b)). Furthermore, Cox regression analysis demonstrated that no baseline parameter was a significant predictor of virological response (Table 3).

### 3.4. HBeAg Seroconversion

After treatment with ETV and TDF, 5/66 (7.6%) and 4/30 (13.3%) patients achieved HBeAg seroconversion, respectively (*p* = 0.60), with median times to seroconversion of 60 and 42 weeks, respectively (*p* = 0.48), indicating no statistically significant differences in HBeAg seroconversion rates and times between the two groups. In agreement, Kaplan-Meier analysis indicated no significant difference in HBeAg seroconversion rates between TDF and ETV treated patients (Figure 1(c)).

### 3.5. Breakthrough and Resistance

No patients in either group developed chemical breakthrough. Only 1 case in the entecavir group developed virological breakthrough at 72 weeks of follow-up. HBV-DNA levels in this patient rebounded to 5.15 log_10_ IU/mL from <100 IU/mL at 60 weeks. However, resistance test was not performed at that time. Adefovir dipivoxil was administered to the patient, and HBV-DNA was not detected at 96 weeks.

### 3.6. Safety and Tolerability

The two drugs were well tolerated, with no report of serious clinical adverse reactions. Serum creatinine was not observed in the TDF group.

## 4. Discussion

The main concern in CHB patients is the disease progressing to decompensated cirrhosis and hepatocellular carcinoma. Circulating serum HBV DNA levels play a central role in disease progression in these patients. Previous large, long-term studies have shown that serum HBV DNA levels are a major risk factor for cirrhosis: the higher the HBV DNA level, the higher the risk of cirrhosis and HCC [11, 12].

The REVEAL-HBV study also supported an approach of maximal and timely suppression of viral replication as the target of therapeutic management of patients with CHB, which may delay or prevent liver disease progression [13]. The emergence of resistant HBV is related to viral load at the onset of therapy. Previous studies have indicated that patients with high pretreatment serum HBV DNA levels seem to be at a higher risk for developing resistant HBV during long-term LAM or ADV therapy [14, 15]. Therefore, current guidelines recommend ETV and TDF as first-line nucleoside/nucleotides. These are potent antivirals that effectively suppress HBV DNA replication with high genetic barrier for resistance [6–8].

Phase III clinical trials have shown that ETV and TDF in treatment-naïve patients suppress HBV DNA to undetectable levels by 48 weeks in 67% and 76% HBeAg-positive CHB patients, respectively [16, 17]. Tenofovir and entecavir have been compared for CHB treatment efficacy and safety, but discrepant data have been reported, probably due to the retrospective design of these studies. Indeed, a multicenter, retrospective study comparing ETV and TDF efficacy for initial treatment of CHB showed no difference between the two patient groups in viral response [18]. Meanwhile, another trial suggested that TDF yields a better virological response compared with ETV (OR 1.796, *p* = 0.01) [19]. The present study aimed to compare complete virological response rates between ETV and TDF in the treatment of HBeAg positive CHB patients with high viral load. As shown above, no statistically significant differences were obtained in the inhibitory effects of the two drugs.

However, a recent report demonstrated that TDF is superior to ETV in achieving complete viral suppression in HBeAg-positive CHB patients with high HBV DNA among HBeAg-positive patients. There was no significant difference in viral suppression between the two drugs among HBeAg-negative patients [20].

During follow-up, very few HBeAg negative CHB patients had high viral load in this study, and were not included in the analyses. Previously reported HBV DNA suppression rates range from 68% to 90% and 61% to 92% for the TDF and ETV groups, respectively, after 48 weeks of therapy [21–23]. In the present study, TDF had higher 48 w virological response (96.7% versus 86.4%). However, 72 weeks of treatment with both drugs resulted in higher virological response rates, with 93.9% and 96.7% obtained for the ETV and TDF groups, respectively. Interestingly, no significant difference was found in virological response between the ETV and TDF groups (96.9% versus 96.7%) at 96 weeks. Moreover, HBV-DNA levels were similar at 4, 12, 24, and 48 weeks between the two groups. These data indicated that the two drugs have similar strengths in inhibiting HBV DNA, in agreement with a recently published meta-analysis assessing 7 studies and showing that ETV and TDF are similar in maintenance HBV DNA inhibition [24].

Serum ALT levels reflect the host immune response to the virus. Virological response is therefore often accompanied by ALT normalization, showing attenuated liver damage. Our study showed that patients in the ETV group had lower serum ALT levels in the early stage of treatment (12 w and 24 w). However, no significant difference in ALT levels was observed between the two groups at any follow-up point, corroborating previous research [25]. Another measure of efficacy is loss of HBeAg and development of antibodies to HBeAg (anti-HBe), referred to as HBeAg seroconversion, which is associated with low HBV-DNA levels and clinical remission of liver disease in most patients. HBeAg seroconversion is also associated with a sustained reduction in HBV-DNA levels [26]. At the time of 72 weeks, HBeAg seroconversion rate is 7.6% for ETV treated patients and 13.3% for TDF treated patients; these values are lower than previously reported. In a meta-analysis, ETV and TDF treatment for 48 weeks yielded similar HBeAg seroconversion rates (10% versus 16%) [24]. Another meta-analysis also showed that among different nucleoside analogues, HBeAg seroconversion rates had no significant difference after 1 year of treatment [27]. HBeAg conversion rates after TDF treatment were 18% and 19% after 2 and 3 years, respectively [28]; meanwhile, a serological conversion rate of 24% was obtained after 2 years of treatment with entecavir [29].

As far as safety is concerned, the two drugs showed good tolerability in this study, in agreement with previous reports. The current guidelines suggest monitoring the renal function during TDF therapy [6–8]. In a large four year study assessing safety of TDF for the treatment of HIV infection, 2% of patients reported increased serum creatinine levels [30].

In a randomized controlled trial of TDF for long-term treatment of CHB, serum creatinine levels remained stable during the 3-year period, with <1% of patients showing a confirmed 0.5 mg/dL increase in creatinine. In our study, TDF did not cause kidney safety problems [28].

A recent cohort study in Hong Kong assessing nucleoside analogues (NA) in treating 53500 CHB patients with median follow-up of 4.9 years found that NA do not increase the risk for renal and bone events [31]. Ha et al. also suggested that TDF is not an independent predictor of severe kidney damage; however, they recommended monitoring closely the renal function during antiviral therapy, especially in the elderly or patients with impaired renal function [32]. Although more than half of the patients in this work have been previously assessed [9], the two study populations are quite different in that only chronic hepatitis B patients with high HBV DNA were reevaluated here, and our current findings complement the previous conclusion [9].

Because of its retrospective design, our research has limitations. In addition, sample size was small and follow-up time was short. Nevertheless, this study is meaningful, since only few reports directly compare the efficacy and safety of ETV and TDF in CHB patients with high viral load.

## 5. Conclusion

This study indicated that ETV and TDF have comparable efficacy in suppressing HBV-DNA replication and are well tolerated in HBeAg-positive nucleos(t)ide-naïve CHB patients with high HBV DNA. However, further randomized prospective studies with more patients are needed to confirm these findings.

## Figures and Tables

**Table 1:** Baseline characteristics of patients administered tenofovir (TDF) or entecavir (ETV).

	ETV (*n* = 66)	TDF (*n* = 30)	*p*
Age, years	35.1 ± 4.5	33.7 ± 4.6	0.28
Gender, male	74.2% (49/66)	76.7% (23/30)	1.00
Height (cm)	167.09 ± 7.22	164.93 ± 7.45	0.19
Weight (kg)	62.58 ± 10.43	58.37 ± 7.98	0.05
History of alcohol use	21.2% (14/66)	26.7% (8/30)	0.61
History of smoking	19.7% (13/66)	26.7% (8/30)	0.44
Family history of Hepatitis B	71.2% (47/66)	70.0% (21/30)	1.00
Pretreatment HBV-DNA, log_10_ IU/mL	7.33 ± 0.79	7.25 ± 0.83	0.62
Pretreatment serum ALT, U/L	154.59 ± 122.05	168.53 ± 112.39	0.60
Elevated serum ALT before therapy	93.9% (62/66)	96.7% (29/30)	0.58

IU/mL, international unit per milliliter; U/L, unit per liter; ALT, alanine aminotransferase.

**Table 2:** Cumulative virological responses in patients with chronic hepatitis B.

Undetectable HBV-DNA (%)	ETV (*n* = 66)	TDF (*n* = 30)	*p*
4 weeks	4.8% (3/66)	0% (0/30)	0.60
12 weeks	34.8% (23/66)	43.3% (13/30)	0.43
24 weeks	56.1% (37/66)	63.3% (19/30)	0.50
36 weeks	69.7% (46/66)	90% (27/30)	0.03
48 weeks	86.4% (57/66)	96.7% (29/30)	0.13
72 weeks	93.9% (62/66)	96.7% (29/30)	0.58
96 weeks	96.9% (63/65^#^)	96.7% (29/30)	0.58

TDF, tenofovir; ETV, entecavir; ^#^one patient was excluded because of adding adefovir for the virological breakthrough.

**Table 3:** Cox regression analysis identifying independent variables predictive of virological response.

Variables	HR (95% CI)	*p*
Gender	0.522 (0.273–0.997)	0.07
Age	1.005 (0.966–1.045)	0.81
Height	1.018 (0.974–1.063)	0.43
Weight	0.994 (0.965–1.024)	0.68
Alcohol history	1.158 (0.677–1.983)	0.59
Smoking history	0.967 (0.558–1.675)	0.90
Family history	1.739 (1.083–2.794)	0.07
ALT baseline (U/L)	1.000 (0.998–1.002)	0.84
HBV DNA baseline (log_10_ IU/mL)	0.904 (0.699–1.169)	0.44
Therapy with TDF versus ETV	0.896 (0.552–1.457)	0.66

TDF, tenofovir; ETV, entecavir; ALT, alanine aminotransferase; IU/mL, international unit per milliliter; U/L, unit per liter; HR, hazard ratio; CI, confidence interval.
